# Medication for Opioid Use Disorder After Serious Injection-Related Infections in Massachusetts

**DOI:** 10.1001/jamanetworkopen.2024.21740

**Published:** 2024-07-24

**Authors:** Simeon D. Kimmel, Alexander Y. Walley, Laura F. White, Shapei Yan, Christine Grella, Adam Majeski, Michael D. Stein, Amy Bettano, Dana Bernson, Mari-Lynn Drainoni, Jeffrey H. Samet, Marc R. Larochelle

**Affiliations:** 1 Section of General Internal Medicine, Department of Medicine, Boston University Chobanian & Avedisian School of Medicine and Boston Medical Center, Boston, Massachusetts; 2 Section of Infectious Diseases, Department of Medicine, Boston University Chobanian & Avedisian School of Medicine and Boston Medical Center, Boston, Massachusetts; 3 Department of Biostatistics, Boston University School of Public Health, Boston, Massachusetts; 4 Semel Institute of Neuroscience and Human Behavior, David Geffen School of Medicine, University of California, Los Angeles; 5 Lighthouse Institute, Chestnut Health Systems, Chicago, Illinois; 6 Department of Health, Law and Policy, Boston University School of Public Health, Boston, Massachusetts; 7 Office of Population Health, Department of Public Health, Commonwealth of Massachusetts, Boston; 8 Evans Center for Implementation and Improvement Sciences, Boston University, Boston, Massachusetts

## Abstract

**Importance:**

Serious injection-related infections (SIRIs) cause significant morbidity and mortality. Medication for opioid use disorder (MOUD) improves outcomes but is underused. Understanding MOUD treatment after SIRIs could inform interventions to close this gap.

**Objectives:**

To examine rehospitalization, death rates, and MOUD receipt for individuals with SIRIs and to assess characteristics associated with MOUD receipt.

**Design, Setting, and Participants:**

This retrospective cohort study used the Massachusetts Public Health Data Warehouse, which included all individuals with a claim in the All-Payer Claims Database and is linked to individual-level data from multiple government agencies, to assess individuals aged 18 to 64 years with opioid use disorder and hospitalization for endocarditis, osteomyelitis, epidural abscess, septic arthritis, or bloodstream infection (ie, SIRI) between July 1, 2014, and December 31, 2019. Data analysis was performed from November 2021 to May 2023.

**Exposure:**

Demographic and clinical factors potentially associated with posthospitalization MOUD receipt.

**Main Outcomes and Measures:**

The main outcome was MOUD receipt measured weekly in the 12 months after hospitalization. We used zero-inflated negative binomial regression to examine characteristics associated with any MOUD receipt and rates of treatment in the 12 months after hospitalization. Secondary outcomes were receipt of any buprenorphine formulation, methadone, and extended-release naltrexone examined individually.

**Results:**

Among 9757 individuals (mean [SD] age, 43.3 [12.1] years; 5701 [58.4%] male) who survived a SIRI hospitalization, 4652 (47.7%) received MOUD, 6518 (66.8%) were rehospitalized, and 1068 (10.9%) died within 12 months. Of those treated with MOUD in the 12 months after hospitalization, the mean (SD) number of MOUD initiations during follow-up was 3.0 (1.8), with 985 of 4652 individuals (21.2%) receiving treatment at least 80% of the time. MOUD treatment after SIRI hospitalization was significantly associated with MOUD in the prior 6 months (buprenorphine: adjusted odds ratio [AOR], 16.63; 95% CI, 14.04-19.71; methadone: AOR, 28.61; 95% CI, 22.80-35.90; or naltrexone: AOR, 2.05; 95% CI, 1.58-2.66). Prior buprenorphine (incident rate ratio [IRR], 1.16; 95% CI, 1.09-1.22) or methadone (IRR, 1.90; 95% CI, 1.79-2.02) use was associated with higher treatment rates after hospitalization, and prior naltrexone use (IRR, 0.85; 95% CI, 0.77-0.95) was associated with lower rates.

**Conclusions and Relevance:**

This study found that in the year after a SIRI hospitalization in Massachusetts, mortality and rehospitalization were common, and only half of patients received MOUD. Treatment with MOUD before a SIRI was associated with posthospitalization MOUD initiation and time receiving MOUD. Efforts are needed to initiate MOUD treatment during SIRI hospitalizations and subsequently retain patients in treatment.

## Key Points

**Question:**

After serious injection-related infections (SIRIs), what characteristics are associated with receipt of medication for opioid use disorder (MOUD)?

**Findings:**

In this cohort study of 9757 individuals who survived a SIRI hospitalization in Massachusetts, 47.7% received MOUD in the 12 months after discharge. Treatment with MOUD before the SIRI hospitalization was associated with both receipt of any MOUD treatment and treatment rates in the 12 months after a SIRI hospitalization.

**Meaning:**

In this study, MOUD was underused and prior MOUD treatment was associated with subsequent treatment after SIRI hospitalization.

## Teaser

This cohort study of individuals who survived a serious injection-related infection hospitalization in Massachusetts examines treatment with medications for opioid use disorder and its association with demographic and clinical characteristics.

## Introduction

Serious injection-related infections (SIRIs), including endocarditis, osteomyelitis, epidural abscess, septic arthritis, and bloodstream infections, have more than doubled in the past 2 decades, causing morbidity and mortality for people who inject drugs.^1,2,3^ Most SIRIs are associated with opioid use disorder (OUD), despite regional variation.^4,5,6^ One-year mortality rates are elevated after SIRIs, reaching nearly 30% for endocarditis.^2,7,8,9,10,11,12,13^ Typically, SIRIs are treated with 2 or more weeks of intravenous antibiotics in hospitals, at post–acute care facilities, or at home, costing the health system more than $700 million annually.^14,15^

Although individuals with SIRIs seek care for their infections, hospitalizations are opportunities to initiate medications for opioid use disorder (MOUD).^16,17,18,19^ Methadone and buprenorphine, 2 types of MOUD, are associated with decreased opioid overdose and all-cause mortality.^20^ After SIRIs, MOUD treatment is associated with decreased rehospitalizations and mortality^7,13,20,21,22,23,24^ but are underused and with benefits attenuated by poor retention.^7,21,25^ Thus, there is an urgent need to understand how to improve MOUD initiation and retention after hospital discharge.

With an OUD prevalence of nearly 5%, one of the highest opioid-related hospitalization rates in the country, and a tradition of MOUD delivery,^26,27,28,29,30^ Massachusetts is a suitable site to investigate MOUD treatment after SIRIs.^31^ We used the Massachusetts Public Health Data Warehouse (PHD) to identify individuals with OUD hospitalized for SIRIs and examined MOUD receipt and treatment rates in the 12 months after discharge.^32^ We sought to describe MOUD treatment and its association with demographic and clinical characteristics to inform efforts to improve care. Identifying disparities by race, sex, housing status, and MOUD history or disposition, for example, may shape interventions to improve MOUD uptake and engagement.

## Methods

### Study Design and Data Source

This retrospective cohort study used the Massachusetts PHD, which included all Massachusetts residents with a claim in the All-Payer Claims Database (APCD).^33^ The APCD records were linked longitudinally at the individual level to 23 government datasets, 11 of which were used in this study. Studies that use the PHD have examined OUD prevalence and MOUD administration after overdose and endocarditis, among other conditions.^7,31,34,35^ Individual counts smaller than 10 are suppressed for privacy. The Boston Medical Center/Boston University Medical Campus Institutional Review Board determined this study was not human subjects research and therefore waived the need for study approval and informed consent. We followed the Strengthening the Reporting of Observational Studies in Epidemiology (STROBE) reporting guidelines.^36^

### Cohort Selection

At the time of this analysis, the PHD included data from January 1, 2014, to December 31, 2020, for the 11 datasets used. The cohort included individuals aged 18 to 64 years with OUD at the time of hospitalization for a serious infection (ie, SIRI) between July 1, 2014, and December 31, 2019, allowing 6 months before the index hospitalization for covariate identification and 12 months of MOUD-receipt assessment (eFigure 1 in Supplement 1). We excluded individuals older than 64 years to limit potential misclassification of infections due to noninjection factors (eg, dialysis and valve disease) and individuals younger than 18 because they receive MOUD and inject opioids less frequently.^37,38^

First, we identified discharges with a serious infection diagnosis, including endocarditis, osteomyelitis, septic arthritis, epidural abscess, or bloodstream infection (eg, bacteremia or fungemia), using *International Classification of Diseases, Ninth Revision* (*ICD-9*) and *International Statistical Classification of Diseases and Related Health Problems, Tenth Revision* (*ICD-10*) (eTable 2 in Supplement 1). Second, we identified individuals whose infections were likely related to opioid injection based on record of OUD in the 6 months before hospitalization based on OUD billing codes, methadone or buprenorphine treatment, admission to a Bureau of Substance Addiction Services (BSAS) treatment facility, or an opioid-related ambulance trip. Buprenorphine, including long-acting formulations, was identified from Prescription Monitoring Program data or procedure codes. Methadone was identified from the BSAS data or procedure codes (eTables 3 and 4 in Supplement 1).

The first qualifying SIRI hospitalization during the study period was defined as the index hospitalization and triggered cohort inclusion. Because the focus was posthospitalization MOUD treatment, we excluded individuals who died during the hospitalization or were discharged to hospice (eFigure 2 in Supplement 1). For those with multiple qualifying hospitalizations, we examined MOUD treatment after the first to preserve individual-level analyses.

### Main Outcomes and Measures

The main outcome was MOUD receipt measured weekly in the year after discharge for a SIRI hospitalization. Any week with at least 1 day of treatment with methadone, buprenorphine, or extended-release naltrexone was considered a week with treatment. Buprenorphine and methadone receipt were identified as described above. We identified extended-release naltrexone from pharmacy or procedure codes in the APCD. We used days’ supply and dispensing data for prescriptions (including long-acting buprenorphine) and date of claim plus 27 days for extended-release naltrexone to classify weeks receiving MOUD. Because the PHD does not include MOUD from inpatient or correctional pharmacies, receipt during hospitalizations or during incarceration could not be examined, and these weeks were excluded.

For secondary analyses, we examined weekly receipt of methadone, buprenorphine, and extended-release naltrexone as individual outcomes in separate models. We also reported the number of MOUD initiations, defined as MOUD receipt during the follow-up period after at least 2 eligible weeks without any MOUD, excluding incarceration or rehospitalization.

For descriptive purposes, we also report on rehospitalization, incarceration, and all-cause and opioid-related mortality from death records. As with previous research in the PHD, opioid-related deaths were determined by the medical examiner or standardized assessment by the Massachusetts Department of Public Health.^35^

### Exposures and Confounders

To describe the cohort and examine associations with MOUD receipt, we included multiple demographic and clinical characteristics. We identified age and insurance status at time of index hospitalization from the APCD. To examine racial disparities in MOUD treatment after SIRIs, data on race and ethnicity were obtained from the APCD and corroborated across multiple data sources in the PHD using a tiered method. We identified anxiety, depression, alcohol use disorder, and stimulant use disorder using *ICD-9* or *ICD-10* codes in the APCD (eTable 4 in Supplement 1) or admission to a BSAS-licensed facility for the relevant disorder. We calculated a modified Elixhauser Comorbidity Index using the presence of 31 comorbidities identified with *ICD-9* or *ICD-10* codes during the index hospitalization or in the prior 6 months.^39^ Because alcohol use, drug use, anxiety, and depression were separate variables in this study, these were excluded from the total score. We identified receipt of the opioid analgesic benzodiazepine in the 6 months before a SIRI discharge using Prescription Monitoring Program data and receipt of a naloxone prescription using pharmacy records. We identified methadone, buprenorphine, and naltrexone (extended-release or oral) in the 6 months before hospitalization as described earlier. We identified homelessness in the 6 months before discharge for the SIRI using multiple sources in the PHD (eTable 6 in Supplement 1). Additionally, we examined hospitalization length and discharge disposition (eg, routine, skilled nursing facility [SNF], or rehabilitation facility) and infection type.

### Statistical Analysis

We used χ^2^ tests to compare baseline cohort characteristics by MOUD receipt in the follow-up period. We calculated the proportion receiving MOUD for weeks leading up to and after the index hospitalization and repeated analyses stratified by discharge home and to a SNF or rehabilitation facility. We calculated the proportion of weeks with MOUD in the 1-year follow-up period, excluding weeks with acute care hospitalization, with incarceration, or after death. We used the Cramer V test to examine correlations of covariates and created a composite variable for anxiety and/or depression based on correlation greater than 0.4. Zero-inflated negative binomial regression is a 2-part model for use in the presence of excess zeros. Because no MOUD receipt was common, we used this approach to examine factors associated with (1) receipt of any MOUD (model 1) and (2) weeks of MOUD receipt in the follow-up period (model 2). In model 2, we included an offset term for number of weeks in which MOUD receipt could be observed (ie, excluding weeks incarcerated, hospitalized, or after death) to model MOUD treatment rates (eFigure 3 in Supplement 1). For secondary analyses, we used the same approach to examine receipt of methadone, buprenorphine, or extended-release naltrexone as individual outcomes. Two-sided *P* < .05 was considered statistically significant. We performed analyses using SAS Studio, version 3.81 (SAS Institute Inc). Data analysis was performed from November 2021 to May 2023.

## Results

We identified 65 723 hospitalizations for serious infections, 19 540 (29.7%) of which included record of OUD during the hospitalization or in the 6 months prior. We excluded 781 hospitalizations resulting in death or hospice discharge. As the first qualifying hospitalization triggered cohort inclusion, 9002 subsequent SIRI hospitalizations were excluded. The resulting study cohort included 9757 individuals (mean [SD] age, 43.3 [12.1] years; 5701 [58.4%] male and 4056 [41.6%] female; 55 [0.6%] American Indian, other non-Hispanic race [no additional description available from the database], or unknown; 39 [0.4%] non-Hispanic Asian or Pacific Islander; 807 [8.3%] non-Hispanic Black; 1239 (12.7) Hispanic; and 7617 [78.1%] non-Hispanic White) with an index SIRI hospitalization (eFigure 2 in Supplement 1). Of these individuals, during 12 months of potential follow-up, 6518 (66.8%) were rehospitalized, 459 (4.7%) were incarcerated in prison, 205 (2.1%) died of opioid overdose, and 1068 (10.9%) died of any cause. Within 3 months of discharge, 4654 (47.7%) were rehospitalized, 166 (1.7%) were incarcerated, and 378 (3.9%) died, including 49 (0.5%) of an opioid overdose.

### MOUD Receipt Before and After SIRI Hospitalization

**Figure 1.**
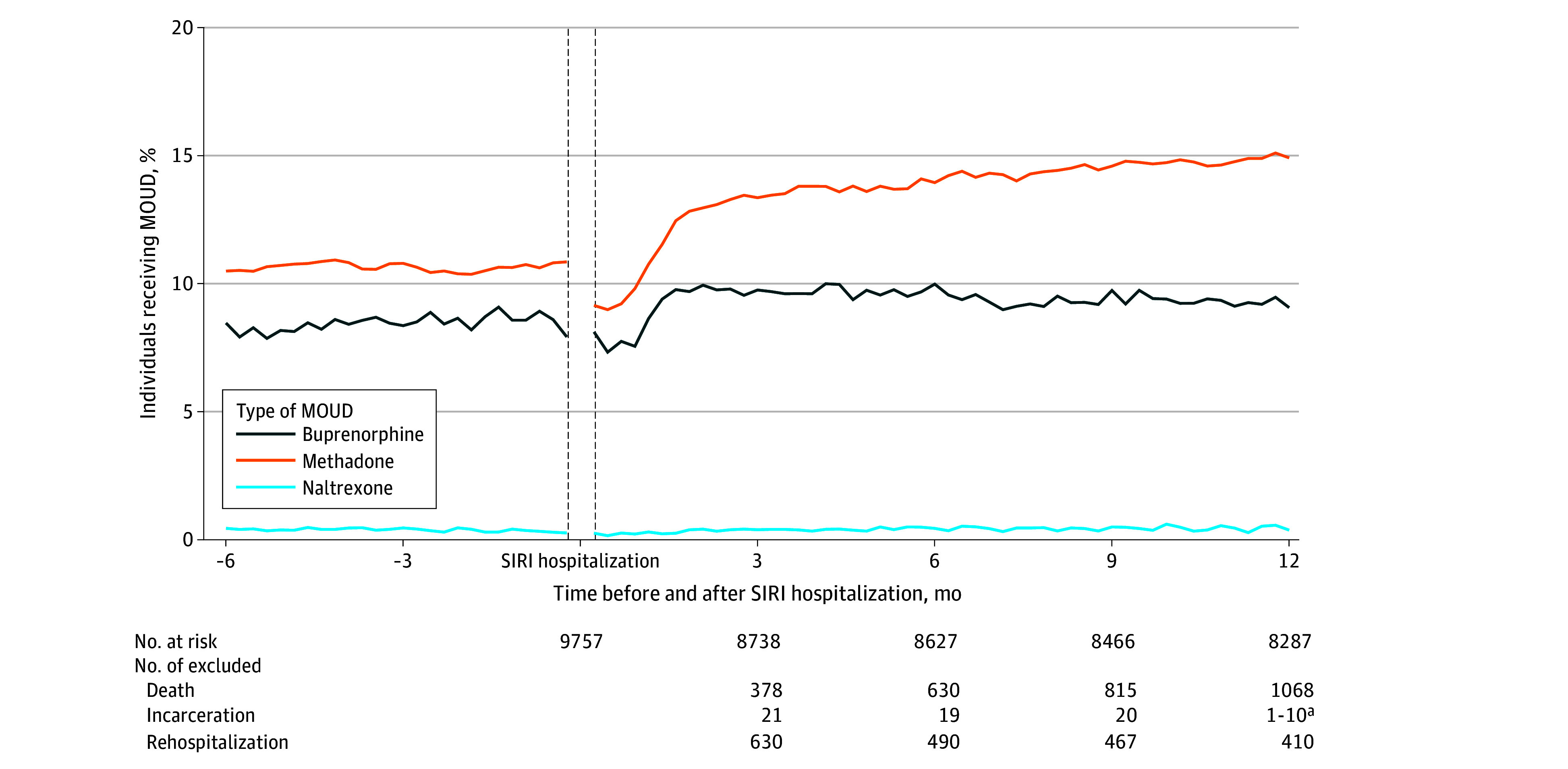
Weekly Methadone, Buprenorphine, and Naltrexone Receipt 6 Months Before and 12 Months After Serious Injection-Related Infection (SIRI) Between January 1, 2014, and December 31, 2019, in Massachusetts Index hospitalization occurred between July 1, 2014, and December 31, 2019. MOUD indicates medication for opioid use disorder. ^a^Suppressed due to count less than 10.

**Figure 2.**
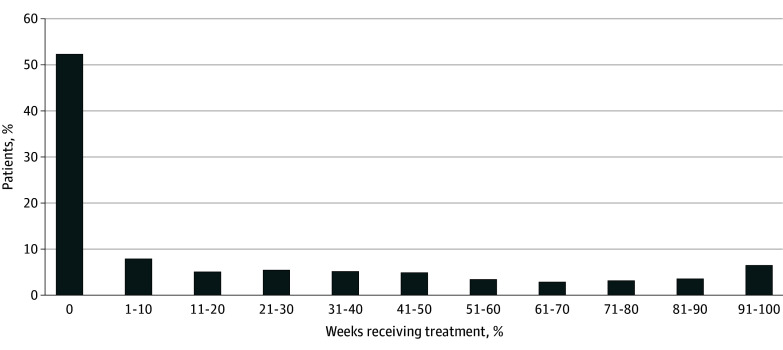
Percentage of Weeks With Receipt of Medication for Opioid Use Disorder (MOUD) After Serious Injection-Related Infection Between July 1, 2014, and December 31, 2019, in Massachusetts Percentage of weeks receiving MOUD treatment was calculated using total weeks of MOUD in the numerator and weeks during which MOUD receipt was observable in our data as the denominator. Weeks while hospitalized, during incarceration, and after death were excluded.

In the 12 months after an index SIRI hospitalization, 4652 of 9757 individuals (47.7%) received any MOUD. Of those who received MOUD, the mean (SD) number of MOUD initiations during follow-up was 3.0 (1.8). In the week before hospitalization, of 9116 individuals, 1735 (19.0%) were treated with MOUD: 722 (7.9%) with buprenorphine, 989 (10.8%) with methadone, and 24 (0.3%) with extended-release naltrexone. Three months after discharge, of 8750 individuals, 2048 (23.4%) were treated with MOUD, with buprenorphine treatment increasing to 835 (9.5%), methadone to 1177 (13.5%), and extended-release naltrexone to 36 (0.4%). At 12 months after discharge, of 8275 individuals, 2015 (24.4%) were treated with MOUD, with buprenorphine treatment decreasing to 750 (9.1%), methadone treatment increasing to 1234 (14.9%), and extended-release naltrexone treatment stable at 31 (0.4%) (Figure 1). Results were similar when stratified by discharge to home or an SNF (eFigure 4 in Supplement 1). Among the 4652 who received MOUD after SIRI hospitalization, 1894 (40.7%) had more than 50% of follow-up while receiving MOUD treatment and 985 (21.2%) had more than 80% of follow-up (Figure 2).

### Participant Characteristics by MOUD Receipt

As indicated in Table 1, those who received MOUD in the 12 months after infection, compared with those not treated with MOUD, were significantly more likely to have received treatment with buprenorphine (1885 [40.5%] vs 349 [6.8%], *P* < .001), methadone (1386 [29.8%] vs 178 [3.5%], *P* < .001), or naltrexone (273 [5.9%] vs 156 [3.1%], *P* < .001) in the 6 months before the SIRI. Additionally, they were more likely to have filled a naloxone prescription (710 [15.3%] vs 316 [6.2%], *P* < .001), more likely have experienced homelessness (1267 [27.2%] vs 749 [14.7%], *P* < .001) or an opioid overdose (690 [14.8%] vs 655 [12.8%], *P* = .004), and less likely to be prescribed opioids (1550 [33.3%] vs 3133 [61.4%], *P* < .001) or benzodiazepines (1256 [27.0%] vs 1709 [33.5%], *P* < .001) in the 6 months before SIRI. Those who received MOUD were more likely young (aged 18-34 years: 1772 [38.1%] vs 1132 [22.2%], *P* < .001), less likely non-Hispanic Black (278 [6.0%] vs 529 [10.4%], *P* < .001), more likely to have Medicaid (3114 [66.9%] vs 2421 [47.4%], *P* < .001), and less likely to have commercial (527 [11.3%] vs 918 [18.0%]) or Medicare insurance (828 [17.8%] vs 1559 [30.5%]) (*P* < .001 for both). They were more likely to have anxiety or depression (3370 [72.4%] vs 3233 [63.3%], *P* < .001), more likely to have alcohol (1594 [34.3%] vs 1536 [30.1%], *P* < .001) or stimulant use disorder (1111 [23.9%] vs 660 [12.9%], *P* < .001), and less likely to have high Elixhauser Comorbidity Index scores (≥3) (2169 [46.6%] vs 3359 [65.8%], *P* < .001).

**Table 1.** Characteristics of Individuals With and Without MOUD Receipt After Serious Injection-Related Infections in Massachusetts, July 1, 2014, to December 31, 2019^a^

Characteristic	Total (N = 9757)	Any MOUD in 12-mo follow-up (n = 4652)	No MOUD in 12-mo follow-up (n = 5105)	*P* value
Age group, y				
18-34	2904 (29.8)	1772 (38.1)	1132 (22.2)	<.001
35-49	3339 (34.2)	1791 (38.5)	1548 (30.3)	
50-64	3514 (36.0)	1089 (23.4)	2425 (47.5)	
Sex				
Male	5701 (58.4)	2644 (56.8)	3057 (59.9)	.002
Female	4056 (41.6)	2008 (43.2)	2048 (40.1)	
Race and ethnicity				
American Indian, other,^b^ or unknown	55 (0.6)	Data suppressed	39 (0.8)	<.001
Asian or Pacific Islander, non-Hispanic	39 (0.4)	Data suppressed	31 (0.6)	
Black, non-Hispanic	807 (8.3)	278 (6.0)	529 (10.4)	
Hispanic	1239 (12.7)	620 (13.3)	619 (12.1)	
White, non-Hispanic	7617 (78.1)	3730 (80.2)	3887 (76.1)	
Insurance				
Commercial	1445 (14.8)	527 (11.3)	918 (18.0)	<.001
Medicaid	5535 (56.7)	3114 (66.9)	2421 (47.4)	
Medicare	2387 (24.5)	828 (17.8)	1559 (30.5)	
Self-pay	248 (24.5)	137 (2.9)	111 (1.8)	
Other or unknown	142 (1.5)	46 (1.0)	96 (1.9)	
Homelessness^c^	2016 (20.7)	1267 (27.2)	749 (14.7)	<.001
Anxiety or depression^c^	6063 (67.7)	3370 (72.4)	3233 (63.3)	<.001
Alcohol use disorder^c^	3130 (32.1)	1594 (34.3)	1536 (30.1)	<.001
Stimulant use disorder^c^	1771 (18.2)	1111 (23.9)	660 (12.9)	<.001
Opioid prescription, prior^c^	4683 (48.0)	1550 (33.3)	3133 (61.4)	<.001
Benzodiazepine prescription, prior^c^	2965 (30.4)	1256 (27.0)	1709 (33.5)	<.001
Naloxone prescription, prior^c^	1026 (10.5)	710 (15.3)	316 (6.2)	<.001
Modified Elixhauser Comorbidity Index score^d^				
0	1044 (10.7)	638 (13.7)	406 (8.0)	<.001
1	1573 (16.1)	931 (20.0)	642 (12.6)	
2	1612 (16.5)	914 (19.7)	698 (13.7)	
≥3	5528 (56.7)	2169 (46.6)	3359 (65.80	
MOUD, prior^c^				
Buprenorphine	2234 (22.9)	1885 (40.5)	349 (6.8)	<.001
Methadone	1564 (16.0)	1386 (29.8)	178 (3.5)	<.001
Naltrexone (oral or extended release)	429 (4.4)	273 (5.9)	156 (3.1)	<.001
Opioid overdose^c^	1345 (13.8)	690 (14.8)	655 (12.8)	.004
Length of hospitalization, median (IQR), d	7 (4-11)	7 (4-11)	7 (4-13)	<.001
Disposition site				
Home or routine discharge	2566 (26.3)	1358 (29.2)	1208 (23.7)	<.001
Home with services	1708 (17.5)	567 (12.2)	1141 (22.4)	
Transferred, acute care hospital	866 (8.9)	436 (9.4)	430 (8.4)	
SNF or rehabilitation facility	2884 (29.6)	1290 (27.7)	1594 (31.2)	
Patient-directed discharge	1333 (13.7)	774 (16.6)	559 (11.0)	
Other	400 (4.1)	227 (4.90	173 (3.4)	
Infection type				
Endocarditis	1707 (17.5)	1037 (22.3)	670 (13.1)	<.001
Epidural abscess	688 (7.1)	342 (7.4)	346 (6.8)	
Septic arthritis	1046 (10.7)	593 (12.8)	453 (8.9)	
Osteomyelitis	1932 (19.8)	761 (16.4)	1171 (22.9)	
Bloodstream infection	4384 (44.9)	1919 (41.3)	2465 (48.3)	

Abbreviations: MOUD, medication for opioid use disorder; SIRI, serious injection-related infection; SNF, skilled nursing facility.

^a^ Data are presented as number (percentage) of patients unless otherwise indicated.

^b^ No additional description available from the database.

^c^ Homeless status; anxiety and depression; alcohol use disorder; stimulant use disorder; prior prescriptions for opioids, benzodiazepine, and naloxone; prior MOUD; and opioid overdose were assessed in the 6 months before SIRI hospitalization. All other characteristics were ascertained at time of hospitalization.

^d^ Modified Elixhauser Comorbidity Index score excludes alcohol, drug use, and anxiety or depression, as these characteristics were examined separately.

### Zero-Inflated Negative Binomial Model Results: Any MOUD Receipt

In adjusted models, several characteristics were significantly associated with receipt of any MOUD treatment in the year after discharge for a SIRI (Table 2): having received buprenorphine (adjusted odds ratio [AOR], 16.63; 95% CI, 14.04-19.71), methadone (AOR, 28.61; 95% CI, 22.80-35.90), or naltrexone (AOR, 2.05; 95% CI, 1.58-2.66) during the 6 months before SIRI; endocarditis (AOR, 1.83; 95% CI, 1.55-2.15) or septic arthritis (AOR, 1.80; 95% CI, 1.48-2.18) compared with a bloodstream infection; having anxiety or depression (AOR, 1.39; 95% CI, 1.22-1.58); having filled a naloxone prescription (AOR, 1.43; 95% CI, 1.17-1.76); experiencing homelessness (AOR, 1.46; 95% CI, 1.25-1.69); or having Medicaid (AOR, 1.56; 95% CI, 1.32-1.85). Characteristics negatively associated with any MOUD receipt included the following: age of 50 to 64 years compared with 18 to 34 years (AOR, 0.51; 95% CI, 0.43-0.60); filling an opioid prescription in the 6 months before the SIRI (AOR, 0.56; 95% CI, 0.49-0.64); non-Hispanic Black race (AOR, 0.72; 95% CI: 0.59-0.89); having an Elixhauser Comorbidity Index score of 3 or more (AOR, 0.62; 95% CI, 0.51-0.76); and discharge home with services (AOR, 0.65; 95% CI, 0.54-0.78), discharge to an SNF or rehabilitation facility (AOR, 0.79; 95% CI, 0.67-0.92), or patient-directed discharge (AOR, 0.76; 95% CI, 0.63-0.92) compared with routine discharge.

**Table 2.** Zero-Inflated Negative Binomial Model Results: Association of Characteristics With Any MOUD Receipt and Weeks Treated With MOUD Among Individuals With Serious Injection-Related Infections, Massachusetts, July 1, 2014, to December 31, 2019^a^

Characteristic	Any MOUD (N = 9757)		Total weeks receiving MOUD (n = 4652)	
	AOR (95% CI)	*P* value	IRR (95% CI)	*P* value
Age group, y				
18-34	1 [Reference]	NA	1 [Reference]	NA
35-49	0.87 (0.75-1.00)	.05	1.09 (1.03-1.15)	.004
50-64	0.51 (0.43-0.60)	<.001	1.06 (0.99-1.11)	.12
Sex				
Female	1.09 (0.96-1.22)	.17	1.01 (0.96-1.06)	.81
Male	1 [Reference]	NA	1 [Reference]	NA
Race and ethnicity				
American Indian, other,^b^ or unknown	0.44 (0.24-0.80)	.01	1.01 (0.72-1.41)	.96
Black non-Hispanic	0.72 (0.59-0.89)	.002	0.94 (0.84-1.04)	.23
Hispanic	1.01 (0.85-1.19)	.90	0.99 (0.92-1.06)	.75
White non-Hispanic	1 [Reference]	NA	1 [Reference]	NA
Insurance				
Commercial	1 [Reference]	NA	1 [Reference]	NA
Medicaid	1.56 (1.32-1.85)	<.001	1.11 (1.02-1.20)	.01
Medicare	0.98 (0.81-1.19)	.82	1.04 (0.95-1.14)	.40
Self-pay	1.15 (0.78-1.68)	.48	1.15 (0.98-1.34)	.09
Other or unknown	0.70 (0.43-1.13)	.14	0.84 (0.65-1.08)	.18
Homelessness^c^	1.46 (1.25-1.69)	<.001	0.94 (0.89-1.00)	.06
Anxiety or depression^c^	1.39 (1.22-1.58)	<.001	1.05 (0.99-1.11)	.10
Alcohol use disorder^c^	0.92 (0.81-1.05)	.21	0.96 (0.91-1.01)	.14
Stimulant use disorder^c^	1.06 (0.9-1.24)	.49	0.97 (0.91-1.03)	.29
Opioid prescription, prior^c^	0.56 (0.49-0.64)	<.001	0.90 (0.86-0.95)	<.001
Benzodiazepine prescription, prior^c^	0.88 (0.77-1.01)	.08	1.03 (0.98-1.10)	.25
Naloxone prescription, prior^c^	1.43 (1.17-1.76)	<.001	1.02 (0.95-1.10)	.50
Modified Elixhauser Comorbidity Index score^d^				
0	1 [Reference]	NA	1 [Reference]	NA
1	0.98 (0.79-1.21)	.85	1.07 (0.98-1.17)	.11
2	0.87 (0.70-1.09)	.22	1.10 (1.01-1.20)	.03
≥3	0.62 (0.51-0.76)	<.001	1.02 (0.94-1.10)	.64
MOUD, prior^c^				
Buprenorphine	16.63 (14.04-19.71)	<.001	1.16 (1.09-1.22)	<.001
Methadone	28.61 (22.80-35.90)	<.001	1.90 (1.79-2.02)	<.001
Naltrexone (oral or extended release)	2.05 (1.58-2.66)	<.001	0.85 (0.77-0.95)	.004
No MOUD	1 [Reference]	NA	1 [Reference]	NA
Opioid overdose^c^	0.98 (0.83-1.16)	.80	0.96 (0.89-1.03)	.25
Disposition site				
Home or routine discharge	1 [Reference]	NA	1 [Reference]	NA
Home with services	0.65 (0.54-0.78)	<.001	1.04 (0.95-1.13)	.39
Transferred, acute care hospital	0.88 (0.71-1.10)	.26	0.96 (0.87-1.05)	.35
SNF or rehabilitation facility	0.79 (0.67-0.92)	.002	0.94 (0.88-1.00)	.07
Patient-directed discharge	0.76 (0.63-0.92)	.004	0.86 (0.80-0.93)	<.001
Other	1.10 (0.83-1.48)	.50	1.02 (0.90-1.15)	.78
Infection type				
Endocarditis	1.83 (1.55-2.15)	<.001	1.03 (0.96-1.10)	.40
Epidural abscess	1.24 (0.98-1.56)	.07	1.05 (0.95-1.16)	.33
Septic arthritis	1.80 (1.48-2.18)	<.001	0.98 (0.91-1.06)	.64
Osteomyelitis	1.05 (0.89-1.23)	.57	1.05 (0.91-1.06)	.64
Bloodstream infection	1 [Reference]	NA	1 [Reference]	NA

Abbreviations: AOR, adjusted odds ratio; IRR, incident rate ratio; MOUD, medication for opioid use disorder; NA, not applicable; SNF, skilled nursing facility.

^a^ Zero-inflated negative binomial models with a 2-step modeling approach. The first step, receipt of any MOUD, is presented as AOR, and the second step, total treatment time, is presented as IRR.

^b^ No additional description available from the database.

^c^ Homeless status; anxiety and depression; alcohol use disorder; stimulant use disorder; prior prescriptions for opioids, benzodiazepine, and naloxone; prior MOUD; and prior opioid overdose were assessed in the 6 months before SIRI hospitalization. All other characteristics were ascertained at time of hospitalization.

^d^ Modified Elixhauser Comorbidity Index score excludes alcohol, drug use, and anxiety or depression because these characteristics were examined separately.

### Zero-Inflated Negative Binomial Model Results: MOUD Treatment Rates

Among the 4652 individuals receiving MOUD treatment, the following characteristics were significantly associated with higher rate ratios of MOUD treatment during follow-up: buprenorphine (incident rate ratio [IRR], 1.16; 95% CI, 1.09-1.22) or methadone (IRR, 1.90; 95% CI, 1.79-2.02) treatment in the 6 months before hospitalization and ages of 35 to 49 years (IRR, 1.09; 95% CI, 1.03-1.15) compared with 18 to 34 years. Characteristics associated with lower treatment rate ratios were treatment with oral or extended-release naltrexone (IRR, 0.85; 95% CI, 0.77-0.95) and opioid prescription (IRR, 0.90; 95% CI, 0.86-0.95) in the 6 months before the index SIRI hospitalization and patient-directed discharge compared with routine discharge (IRR, 0.86; 95% CI, 0.80-0.93).

### Sensitivity Analyses

Analyses with buprenorphine, methadone, and extended-release naltrexone as separate outcomes had similar results (eTables 7-9 in Supplement 1). Notable associations were found for prior treatment with the same class of MOUD, with AORs of 15.94 (95% CI, 13.87-18.32) for buprenorphine, 31.55 (95% CI, 26.69-37.31) for methadone, and 10.38 (95% CI, 7.70-14.00) for naltrexone. In contrast, prior treatment with methadone was negatively associated with any buprenorphine receipt (AOR, 0.60; 95% CI, 0.51-0.72) and lower buprenorphine rate ratios (IRR, 0.84; 95% CI, 0.74-0.95). For models with extended-release naltrexone as the outcome, prior methadone treatment was negatively associated with any naltrexone receipt (AOR, 0.63; 95% CI, 0.43-0.93).

## Discussion

In this cohort of 9757 individuals with OUD and SIRIs, more than half did not receive MOUD in the year after hospitalization despite evidence that these medications reduce rehospitalization and mortality.^7,13,21,22,23^ The period after SIRI hospitalization may be a critical opportunity to deliver substance use treatment, as 1-year mortality was 10.9%. Low MOUD treatment rates after SIRI hospitalization is likely a failure to initiate and link to MOUD treatment during hospitalizations. Improved clinician effort and systems such as multidisciplinary addiction consult services are needed to deliver MOUD to this population during a period when motivation may be high.^16,40,41,42^

Approximately 1 in 4 individuals received MOUD 3 months after discharge, a low percentage but higher than some previous estimates after SIRIs, which range from 5% to 25%.^7,9,21,22^ The characteristic most associated with receiving MOUD in the year after a SIRI was prior MOUD receipt, with strongest associations for methadone.^43,44^ Low-barrier access to MOUD in the community is needed to not only reduce risks in the first place but also improve uptake of MOUD after complications. However, initiation is only one challenge; retention requires greater attention. Only 40% of those who received MOUD after a SIRI were treated for more than half of the subsequent year. Of the 3 MOUD types, only methadone treatment rates increased during follow-up. Methadone regulations may deter some from initiating the medication, but it seems to be associated with improved retention after SIRI.^44,45,46,47^ Long-acting injectable buprenorphine may improve treatment retention after SIRIs, but research is ongoing for this population.^48,49,50^

Several other characteristics were associated with MOUD receipt after discharge: Medicaid, which may provide better MOUD coverage; homelessness, which may increase contact with services with addiction treatment; and anxiety or depression, which may reflect care seeking or likelihood of receiving diagnoses once in care. People with endocarditis and septic arthritis were also more likely to receive posthospitalization MOUD, perhaps reflecting an ability to marshal treatment during lengthier hospitalizations. Programs such as addiction consultations that connect hospitalized people who inject drugs to MOUD treatment are critically needed.^16,19,23,51^

Disposition to SNFs was associated with decreased MOUD receipt, perhaps reflecting admissions that exclude people receiving MOUD or poor linkage after SNF discharge.^52,53^ Being discharged home with services was also associated with less MOUD treatment, which may reflect similar stigma among home service agencies. Patient-directed discharge was associated with decreased receipt of MOUD, perhaps because those not receiving MOUD for withdrawal more commonly leave^54,55,56,57^ or because these discharges are often chaotic, making outpatient linkage challenging.^58^ These findings underscore the importance of improving MOUD delivery for inpatients and after hospitalization.

Finally, non-Hispanic Black patients were less likely to receive MOUD, corroborating the literature about addiction treatment inequities and racism.^59,60,61,62,63^ Because Black individuals are experiencing increasing overdose rates, ensuring access to effective addiction treatment is essential.^64,65^ Multipronged efforts are needed to address interpersonal and structural racism in OUD care.^62,66,67^

Total time receiving MOUD (eg, treatment rates), in addition to initiation or uninterrupted retention, may be an important OUD treatment measure, especially when discontinuation definitions vary.^68^ In this study, treatment rates were lower for the following: those who received opioid analgesics, those who had a patient-directed discharge, and those who received any form of naltrexone before hospitalization. Homelessness was not significantly associated with a lower treatment rate. Nonetheless, because lack of housing presents barriers to medication storage and appointment attendance,^69,70,71^ supportive housing may improve adherence.^72^ Integrating harm reduction, infectious disease and OUD treatment, mobile health, peer support, telephones, and housing assistance may improve MOUD treatment rates.^40,50,73,74,75,76,77,78,79,80^

### Strengths and Limitations 

This study has several strengths, including use of a longitudinal linked dataset,^7,12^ inclusion of all SIRIs in Massachusetts over 4.5 years, and granular weekly MOUD indicators. The modeling approach accounted for treatment interruptions.

This study also has limitations. First, we expected some misclassification of OUD status, including attribution of infections to OUD caused by stimulant injection or other comorbidities. Second, the data do not include MOUD receipt during inpatient hospitalizations or incarceration, limiting assessments of their role in MOUD treatment. In addition, MOUD provided as part of clinical trials was not available, likely affecting few individuals. Third, post–acute care admission dates were not neatly delineated in the PHD. Fourth, this study was subject to residual confounding. We were limited by available data and could not examine all characteristics (eg, methadone dose, transportation, distance to MOUD treatment, and changes to housing). Fifth, generalizability to other locations or times may be limited due to differences in drug supply or treatment landscape.

## Conclusions

Among Massachusetts patients with SIRIs, fewer than 1 in 2 received MOUD in the year after the infection even though these medications are associated with improved mortality and decreased rehospitalization. Among those who received MOUD, only 4 in 10 were treated for at least 50% of the year after hospitalization. Prior MOUD treatment experience was associated with increased MOUD treatment. To improve MOUD initiation and retention after a SIRI, dedicated efforts are needed to integrate MOUD into SIRI follow-up care and reform the addiction treatment delivery system to make services more accessible and appealing.

## Notes

Supplement 1. **eFigure 1.** Cohort construction, covariate, and outcome assessment for individuals with serious injection-related infections (SIRI) **eFigure 2.** Consort diagram, cohort construction **eFigure 3.** Illustrative examples of MOUD assessment following SIRI hospitalization with exclusions for hospitalization, incarceration, and death **eFigure 4.** Weekly methadone, buprenorphine and naltrexone receipt 6 months before and 12 months after hospitalization for serious injection related infection for those discharged home or to skilled nursing facilities or rehabilitation, Massachusetts, January 1, 2014, to December 31, 2020 **eTable 1.** Sources of data in Massachusetts Public Health Data Warehouse in study **eTable 2.** ICD-9 and ICD-10 codes for serious infections **eTable 3.** Identification of opioid use disorder (OUD) **eTable 4.** ICD-9 and ICD-10 codes used to identify opioid use disorder (OUD) **eTable 5.** Codes used to identify covariates **eTable 6.** Datasets used to identify homelessness in the Massachusetts Public Health Data Warehouse **eTable 7.** Zero-inflated negative binomial model results: characteristics associated with any buprenorphine receipt and weeks treated with buprenorphine among individuals with serious injection-related infections, Massachusetts, July 1, 2014, to December 31, 2019 **eTable 8.** Zero-inflated negative binomial model results: characteristics associated with any methadone receipt and weeks treated with methadone among individuals with serious injection-related infections, Massachusetts, July 1, 2014, to December 31, 2019 **eTable 9.** Zero-inflated negative binomial model results: characteristics associated with any extended-release naltrexone receipt and weeks treated with extended-release naltrexone among individuals with serious injection-related infections, Massachusetts, July 1, 2014, to December 31, 2019

Supplement 2.
Data Sharing Statement
