# Errors in Key Points, Abstract, Results, Discussion, Tables, Figures, and Supplement 1

**DOI:** 10.1001/jamanetworkopen.2024.47704

**Published:** 2024-11-01

In the Original Investigation titled “Medication for Opioid Use Disorder After Serious Injection-Related Infections in Massachusetts,”^1^ published July 24, 2024, there were errors in the *ICD-9* and *ICD-10* codes used to identify the cohort of individuals with serious injection-related infections (SIRIs), which resulted in a number of errors related to a change in the cohort size. The overall cohort size has been corrected from 8769 to 9757 individuals, with subsequent corrections to participant characteristics. Corrections to the reported odds ratios and rate ratios have also been made. Notably, these corrections resulted in a decrease in the number of individuals with SIRIs who were experiencing homelessness; a change from no association to significant association between American Indian, other, or unknown race and receipt of medications for opioid use disorder (MOUD); a change from association to no association between stimulant use disorder and MOUD receipt; a change from association to no association between treatment time and homelessness, opioid overdose, and disposition to a skilled nursing facility; and a change from no association to significant association between an Elixhauser score of 2 and treatment time. Relevant corrections have been made to data in the Key Points, abstract, Results, Tables 1 and 2, Figures 1 and 2, and Supplement 1 and to phrasing in the Discussion related to the affected results; however, the key findings and implications of the study remain unchanged. The authors have explained what happened in a Comment,^2^ and this article has been corrected.^1^
