# Supplementary material for: Medication for Opioid Use Disorder After Serious Injection-Related Infections in Massachusetts
Source: JAMA Netw Open. 2024 Jul 24;7(7):e2421740. doi: 10.1001/jamanetworkopen.2024.21740 (PMC11270137; doi:10.1001/jamanetworkopen.2024.21740)
Supplement: Supplement 1. — eFigure 1. Cohort construction, covariate, and outcome assessment for individuals with serious injection-related infections (SIRI) eFigure 2. Consort diagram, cohort construction eFigure 3. Illustrative examples of MOUD assessment following SIRI hospitalization with exclusions for hospitalization, incarceration, and death eFigure 4. Weekly methadone, buprenorphine and naltrexone receipt 6 months before and 12 months after hospitalization for serious injection related infection for those discharged home or to skilled nursing facilities or rehabilitation, Massachusetts, January 1, 2014, to December 31, 2020 eTable 1. Sources of data in Massachusetts Public Health Data Warehouse in study eTable 2. ICD-9 and ICD-10 codes for serious infections eTable 3. Identification of opioid use disorder (OUD) eTable 4. ICD-9 and ICD-10 codes used to identify opioid use disorder (OUD) eTable 5. Codes used to identify covariates eTable 6. Datasets used to identify homelessness in the Massachusetts Public Health Data Warehouse eTable 7. Zero-inflated negative binomial model results: characteristics associated with any buprenorphine receipt and weeks treated with buprenorphine among individuals with serious injection-related infections, Massachusetts, July 1, 2014, to December 31, 2019 eTable 8. Zero-inflated negative binomial model results: characteristics associated with any methadone receipt and weeks treated with methadone among individuals with serious injection-related infections, Massachusetts, July 1, 2014, to December 31, 2019 eTable 9. Zero-inflated negative binomial model results: characteristics associated with any extended-release naltrexone receipt and weeks treated with extended-release naltrexone among individuals with serious injection-related infections, Massachusetts, July 1, 2014, to December 31, 2019 [file jamanetwopen-e2421740-s001.pdf]

Supplementary Online Content

Kimmel SD, Walley AY, White LF, et al. Medications for opioid use disorder after serious injection-related infections in Massachusetts. *JAMA Netw Open*. 2024;7(7):e2421740. doi:10.1001/jamanetworkopen.2024.21740

eFigure 1. Cohort construction, covariate, and outcome assessment for individuals with serious injection-related infections (SIRI)

eFigure 2. Consort Diagram, Cohort Construction

eFigure 3. Illustrative examples of MOUD assessment following SIRI hospitalization with exclusions for hospitalization, incarceration and death

eFigure 4. Weekly Methadone, Buprenorphine and Naltrexone receipt 6 months before and 12 months after hospitalization for serious injection related infection for those discharged home or to skilled nursing facilities or rehabilitation, Massachusetts, Jan 1st, 2014 to December 31st, 2020

eTable 1. Sources of data in Massachusetts Public Health Data Warehouse in study

eTable 2. ICD-9 and ICD-10 Codes for serious infections

eTable 3. Identification of Opioid Use Disorder (OUD)

eTable 4. ICD-9 and ICD-10 Codes used to identify opioid use disorder (OUD)

eTable 5. Codes Used to Identify Covariates

eTable 6. Datasets used to Identify Homelessness in the Massachusetts Public Health Data Warehouse

eTable 7. Zero-inflated negative binomial model results: Characteristics associated with any buprenorphine receipt and weeks treated with buprenorphine among individuals with serious injection-related infections, Massachusetts, July 1st, 2014 to December 31st 2019

eTable 8. Zero-inflated negative binomial model results: Characteristics associated with any methadone receipt and weeks treated with methadone among individuals with serious injection-related infections, Massachusetts, July 1st, 2014 to December 31st 2019

eTable 9. Zero-inflated negative binomial model results: Characteristics associated with any extended-release naltrexone receipt and weeks treated with extended-release naltrexone among individuals with serious injection-related infections, Massachusetts, July 1st, 2014 to December 31st 2019

This supplementary material has been provided by the authors to give readers additional information about their work.

© 2024 Kimmel SD et al. *JAMA Network Open.*

eFigure 1: Cohort construction, covariate, and outcome assessment for individuals with serious injection-related infections (SIRI)

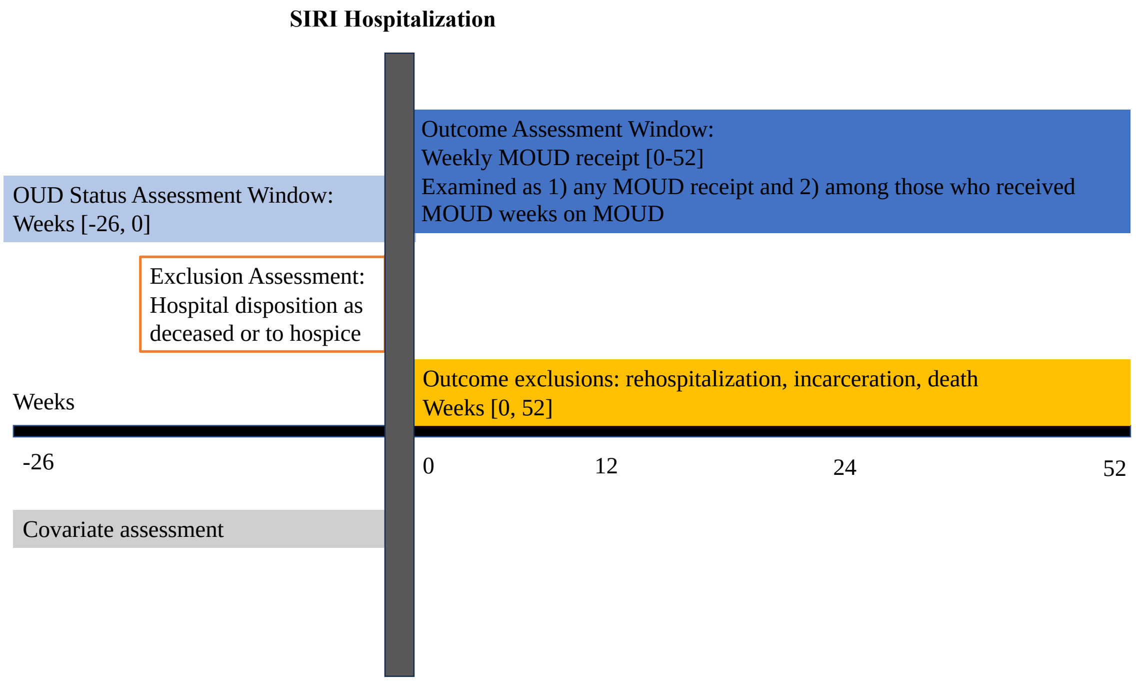

© 2024 Kimmel SD et al. *JAMA Network Open.*

eFigure 2: Consort Diagram, Cohort Construction

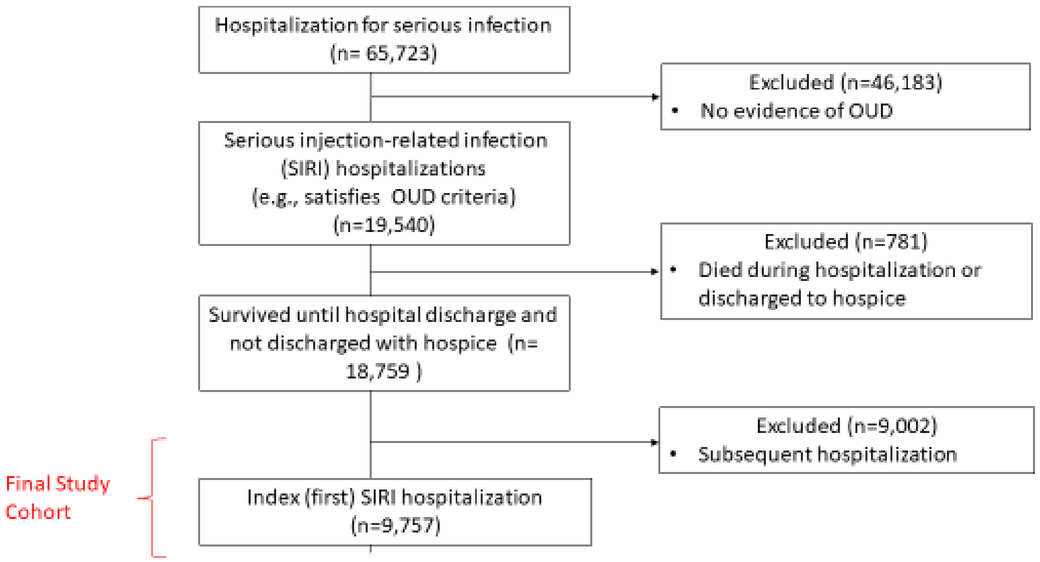

© 2024 Kimmel SD et al. *JAMA Network Open.*

eFigure 3: Illustrative examples of MOUD assessment following SIRI hospitalization with exclusions for hospitalization, incarceration and death

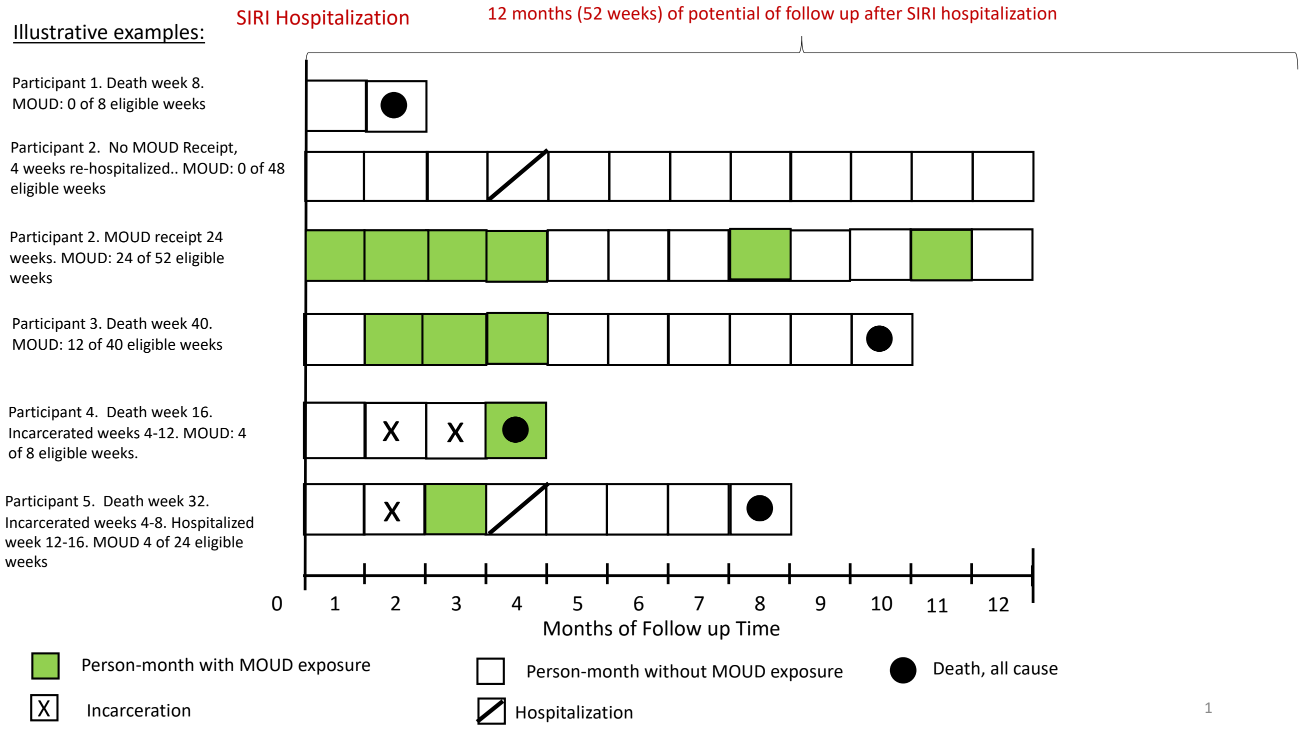

1

© 2024 Kimmel SD et al. *JAMA Network Open.*

eFigure 4: Weekly Methadone, Buprenorphine and Naltrexone receipt 6 months before and 12 months after hospitalization for serious injection related infection for those discharged home or to skilled nursing facilities or rehabilitation, Massachusetts, Jan 1st, 2014 to December 31st, 2020[1,2]

Panel A: Home/Routine Discharge

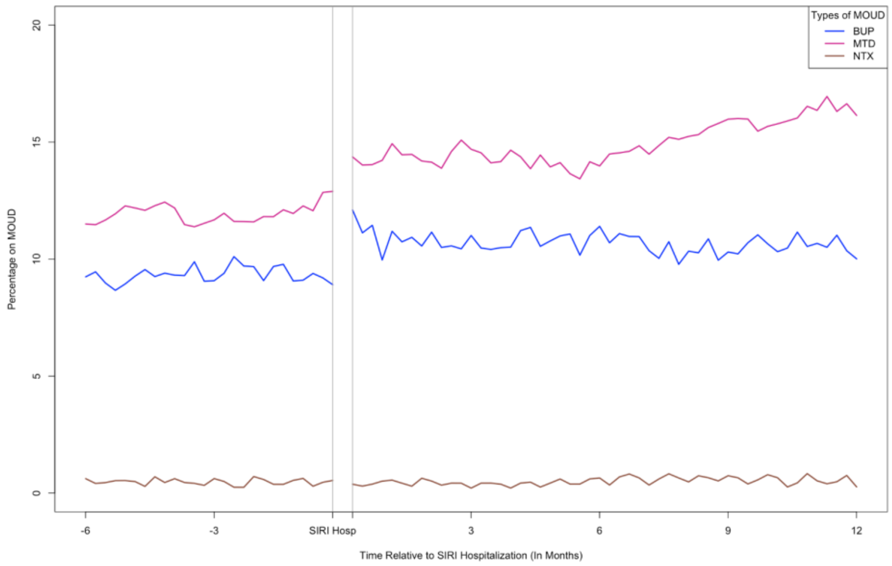

Panel B: Discharge to skilled nursing facility or rehabilitation

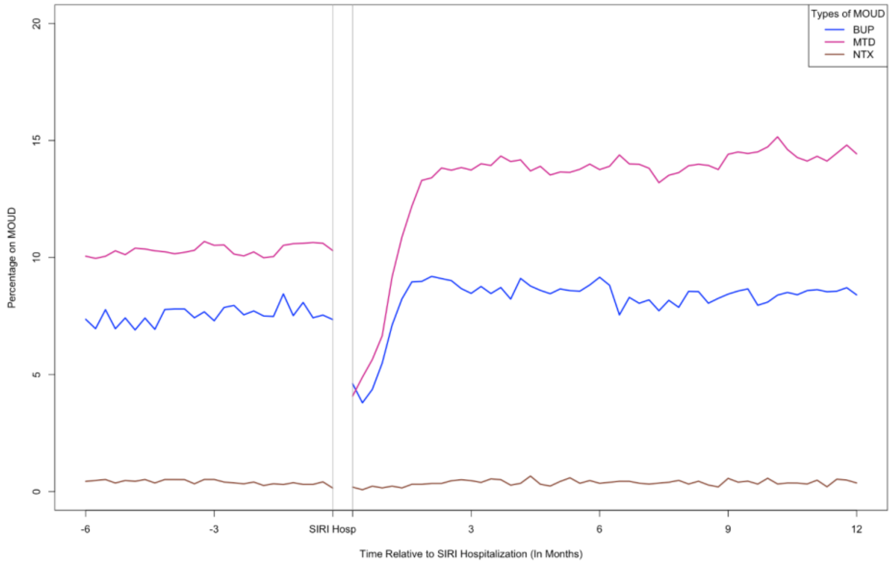

© 2024 Kimmel SD et al. *JAMA Network Open.*

eTable 1. Sources of data in Massachusetts Public Health Data Warehouse in study

All-Payer Claims Database
Registry of Vital Records and Statistics
Prescription Monitoring Program
Acute Care Hospital Case Mix
Bureau of Substance Addiction Services
Department of Mental Health
Department of Housing and Community Development
Massachusetts Ambulance Trip Record Information System
Department of Correction
HIV Incidence
Women, Infants and Children Nutrition Program data

eTable 2. ICD-9 and ICD-10 Codes for serious infections

Endocarditis (ICD-9 Codes)	
036.42	Meningococcal endocarditis
098.84	Gonococcal endocarditis
112.81	Candidal Endocarditis
115.04	Infection by Histoplasma capsulatum endocarditis
115.14	Infection by Histoplasma duboisii endocarditis
115.94	Histoplasmosis, unspecified endocarditis
421.0	Acute and subacute bacterial endocarditis
421.1	Acute and subacute infective endocarditis in diseases classified elsewhere
421.9	Acute endocarditis, unspecified
Endocarditis (ICD-10 Codes)	
B37.6	Candidal endocarditis
I33.0	Acute and subacute infective endocarditis
I33.9	Acute and subacute endocarditis, unspecified
I38	Endocarditis, valve unspecified
I39	Endocarditis and heart valve disorders in diseases classified elsewhere
Osteomyelitis (ICD-9 Codes)	
003.24	Salmonella osteomyelitis
730.00	Osteomyelitis, periostitis, and other infections involving bone, site unspecified
730.01	Osteomyelitis, periostitis, and other infections involving bone, site, shoulder region
730.02	Osteomyelitis, periostitis, and other infections involving bone, site, upper arm
730.03	Osteomyelitis, periostitis, and other infections involving bone, site, forearm
730.04	Osteomyelitis, periostitis, and other infections involving bone, site, hand
730.05	Osteomyelitis, periostitis, and other infections involving bone, site, pelvic region and thigh
730.06	Osteomyelitis, periostitis, and other infections involving bone, site, lower leg

© 2024 Kimmel SD et al. *JAMA Network Open.*

730.07	Osteomyelitis, periostitis, and other infections involving bone, site, ankle and foot
730.08	Osteomyelitis, periostitis, and other infections involving bone, site, other specified sites
730.09	Osteomyelitis, periostitis, and other infections involving bone, site, multiple sites
730.10	Chronic osteomyelitis, site unspecified
730.11	Chronic osteomyelitis, shoulder region
730.12	Chronic osteomyelitis, upper arm
730.13	Chronic osteomyelitis, forearm
730.14	Chronic osteomyelitis, hand
730.15	Chronic osteomyelitis, pelvic region and thigh
730.16	Chronic osteomyelitis, lower leg
730.17	Chronic osteomyelitis, ankle and foot
730.18	Chronic osteomyelitis, other specified sites
730.19	Chronic osteomyelitis, multiple sites
730.20	Unspecified osteomyelitis, site unspecified
730.21	Unspecified osteomyelitis, shoulder region
730.22	Unspecified osteomyelitis, upper arm
730.23	Unspecified osteomyelitis, forearm
730.24	Unspecified osteomyelitis, hand
730.25	Unspecified osteomyelitis, pelvic region and thigh
730.26	Unspecified osteomyelitis, lower leg
730.27	Unspecified osteomyelitis, ankle and foot
730.28	Unspecified osteomyelitis, other specified sites
730.29	Unspecified osteomyelitis, multiple sites
730.81	Other infections involving bone in disease classified elsewhere, shoulder region
730.82	Other infections involving bone in disease classified elsewhere, upper arm
730.83	Other infections involving bone in disease classified elsewhere forearm
730.84	Other infections involving bone in disease classified elsewhere hand
730.85	Other infections involving bone in disease classified elsewhere, pelvic region and thigh
730.86	Other infections involving bone in disease classified elsewhere, lower leg
730.87	Other infections involving bone in disease classified elsewhere, ankle and foot
730.88	Other infections involving bone in disease classified elsewhere, other specified sites
730.89	Other infections involving bone in disease classified elsewhere, multiple sites
730.90	Unspecified infection of bone, site unspecified
730.91	Unspecified infection of bone, shoulder region
730.92	Unspecified infection of bone, upper arm
730.93	Unspecified infection of bone, forearm
730.94	Unspecified infection of bone, hand
730.95	Unspecified infection of bone, pelvic region and thigh
730.96	Unspecified infection of bone, lower leg
730.97	Unspecified infection of bone, ankle and foot
730.98	Unspecified infection of bone, other specified sites
730.99	Unspecified infection of bone, multiple sites

© 2024 Kimmel SD et al. *JAMA Network Open.*

Osteomyelitis (ICD-10 Codes)	
M46.2x	Osteomyelitis of vertebra, site unspecified
M86X	Osteomyelitis
M86.8X	Other osteomyelitis
A0224	Salmonella osteomyelitis
Septic arthritis (ICD-9)	
003.23	Salmonella arthritis
036.82	Meningococcal arthropathy
711.00	Pyogenic arthritis, site unspecified
711.01	Pyogenic arthritis, shoulder region
711.02	Pyogenic arthritis, upper arm
711.03	Pyogenic arthritis, forearm
711.04	Pyogenic arthritis, hand
711.05	Pyogenic arthritis, pelvic region and thigh
711.06	Pyogenic arthritis, lower leg
711.07	Pyogenic arthritis, ankle and foot
711.08	Pyogenic arthritis, other specified sites
711.09	Pyogenic arthritis, multiple sites
711.40	Arthropathy associated with other bacterial diseases, site unspecified
711.41	Arthropathy associated with other bacterial diseases, shoulder region
711.42	Arthropathy associated with other bacterial diseases, upper arm
711.43	Arthropathy associated with other bacterial diseases, forearm
711.44	Arthropathy associated with other bacterial diseases, hand
711.45	Arthropathy associated with other bacterial diseases, pelvic region and thigh
711.46	Arthropathy associated with other bacterial diseases, lower leg
711.47	Arthropathy associated with other bacterial diseases, ankle and foot
711.48	Arthropathy associated with other bacterial diseases, other specified sites
711.49	Arthropathy associated with other bacterial diseases, other specified sites
711.60	Arthropathy associated with mycoses, site unspecified
711.61	Arthropathy associated with mycoses, shoulder region
711.62	Arthropathy associated with mycoses, upper arm
711.63	Arthropathy associated with mycoses, forearm
711.64	Arthropathy associated with mycoses, hand
711.65	Arthropathy associated with mycoses, pelvic region and thigh
711.66	Arthropathy associated with mycoses, lower leg
711.67	Arthropathy associated with mycoses, ankle and foot

© 2024 Kimmel SD et al. *JAMA Network Open.*

711.68	Arthropathy associated with mycoses, other specified sites
711.69	Arthropathy associated with mycoses, multiple sites
711.90	Unspecified infective arthritis, site unspecified
711.91	Unspecified infective arthritis, shoulder region
711.92	Unspecified infective arthritis, upper arm
711.93	Unspecified infective arthritis, forearm
711.94	Unspecified infective arthritis, hand
711.95	Unspecified infective arthritis, pelvic region and thigh
711.96	Unspecified infective arthritis, lower leg
711.97	Unspecified infective arthritis, ankle and foot
711.98	Unspecified infective arthritis, other specified sites
711.99	Unspecified infective arthritis, multiple sites
Septic Arthritis (ICD-10)	
M00.0x	Pyogenic arthritis
M00.8x	Arthritis and polyarthritis due to other bacteria
M00.9	Pyogenic arthritis, unspecified
A0223	Salmonella arthritis
A3983	Meningococcal arthritis
Epidural Abscess (ICD9)	
324.00	Intracranial abscess
324.10	Intraspinal abscess
324.90	Intracranial and intraspinal abscess of unspecified site
Epidural Abscess (ICD10)	
G06.0	Intracranial abscess and granuloma
G06.1	Intraspinal abscess and granuloma
G06.2	Extradural and subdural abscess, unspecified
Bloodstream infection (ICD9)	
038.00	Streptoccocal septicemia
038.10	Staphylococcal septicemia
038.11	Methicillin susceptible Staphylococcus aureus septicemia
038.12	Methicillin resistant Staphylococcus aureus septicemia
038.19	Other staphylococcal septicemia
038.20	Pneumococcal septicemia

© 2024 Kimmel SD et al. *JAMA Network Open.*

038.30	Septicemia due to anaerobes
038.40	Septicemia due to gram-negative organism, unspecified
038.41	Septicemia due to hemophilus influenzae
038.42	Septicemia due to escherichia coli
038.43	Septicemia due to pseudomonas
038.44	Septicemia due to serratia
038.49	Other septicemia due to gram-negative organisms
038.80	Other specified septicemias
415.12	Septic pulmonary embolism
422.92	Septic myocarditis
449.00	Septic arterial embolism
790.70	Bacteremia
Bloodstream infection (ICD10)	
A41.0x	Sepsis due to Methicillin susceptible Staphylococcus aureus
A41.1	Sepsis due to other specified staphylococcus
A41.2	Sepsis due to Methicillin resistant Staphylococcus aureus
A41.3	Sepsis due to Hemophilus influenzae
A41.4	Sepsis due to anaerobes
A41.5X	Sepsis due to other Gram-negative organisms
A41.81	Sepsis due to Enterococcus
A49.9	Bacterial infection, unspecified
R78.81	Bacteremia
B37.7	Candidal sepsis
A40.0	Sepsis due to streptococcus, group A
A40.1	Sepsis due to streptococcus, group B
A40.3	Sepsis due to Streptococcus pneumoniae
A40.8	Other streptococcal sepsis
A40.9	Streptococcal sepsis, unspecified
A41.89	Other specified sepsis
I26.01	Septic pulmonary embolism with acute cor pulmonale
I26.90	Septic pulmonary embolism without acute cor pulmonale

eTable 3. Identification of Opioid Use Disorder (OUD)

OUD Diagnosis	ICD-9 or ICD-10 codes for OUD or opioid overdose in APCD (Supplemental Table 3)
Methadone[1]	HCPCS, CPT, & ICD-10 Procedure Codes in APCD (H0020, G2067, G2078, S0109; HZ91ZZZ, HZ81ZZZ) or methadone treatment in Bureau of Substance Addiction Services data

© 2024 Kimmel SD et al. *JAMA Network Open.*

Buprenorphine	Prescription Monitoring Program record or HCPCS or CPT code for buprenorphine (J0592, G2068, G2069, G2070, G2071, G2072, G2079, J0570-J0575)
Inpatient OUD Treatment	Admission to a Bureau of Substance Addiction Services licensed facility (e.g., detoxification or residential treatment) for opioid use disorder
Opioid-related incident	Massachusetts Ambulance Trip Record Information System

[1]The primary outcome definition included definitions above for methadone, buprenorphine as well as extended release naltrexone codes noted in Supplemental Table 5.

eTable 4. ICD-9 and ICD-10 Codes used to identify opioid use disorder (OUD)

ICD9 Codes for Opioid Use Disorder in All Payer Claims Database	
304.00	Opioid type dependence, unspecified
304.01	Opioid type dependence, continuous
304.02	Opioid type dependence, episodic
304.03	Opioid type dependence, in remission
304.70	Combinations of opioid type drug with any other drug dependence, unspecified
304.71	Combinations of opioid type drug with any other drug dependence, continuous
304.72	Combinations of opioid type drug with any other drug dependence, episodic
304.73	Combinations of opioid type drug with any other drug dependence, in remission
305.50	Opioid abuse, unspecified
305.51	Opioid abuse, continuous
305.52	Opioid abuse, episodic
305.53	Opioid abuse, in remission
965.00	Poisoning by opium
965.01	Poisoning by heroin
965.02	Poisoning by methadone
965.09	Poisoning by opiates and related narcotics
E85.0	Accidental poisoning by heroin
E85.01	Accidental poisoning by methadone
E85.02	Accidental poisoning by opiates and related narcotics
292.0	Drug withdrawal
648.33	Drug dependence antepartum
E935.0	Adverse effect heroin
E935.1	Adverse effect methadone
E935.2	Adverse effect opiates
970.1	Poisoning opioid antagonist
E94.01	Adverse effect opioid antagonist
ICD10 Codes for Opioid Use Disorder in All Payer Claims Database	
F11.X	Opioid related disorders
T40.0X	Poisoning by Opium
T40.1X	Poisoning by Heroin
T40.2X	Poisoning, other opioid
T40.3X	Poisoning, methadone
T40.4X	Poisoning, synthetic narcotics
T40.6X	Poisoning, other narcotics

© 2024 Kimmel SD et al. *JAMA Network Open.*

eTable 5. Codes Used to Identify Covariates

Anxiety	
ICD-9	300.0, 300.01, 300.02, 300.09, 300.20, 300.21, 300.22, 300.23, 300.29, 300.9
ICD-10	F40.X, F41.X
Depression	
ICD-9	296.99, 296.36, 296.35, 296.31, 296.32, 296.33, 296.30, 296.34, 296.26, 296.25, 296.21, 296.22, 296.23, 296.20, 296.24, 311<br>300.4, 625.4
ICD-10	F32.X, F33.X, F34.X, F39.X
Alcohol Use Disorder	
ICD-9	291.1, 291.2, 291.5, 291.81, 291.82, 291.89, 291.9, 303.90, 303.91, 303.92, 303.93, 305.00, 305.01, 305.02, 305.03, V11.3
ICD-10	F10.X
Stimulant Use Disorder	
ICD-9	304.21, 304.22,304.23, 305.60, 305.61, 305.62, 305.63, 304.40, 304.41, 304.42, 969.70, 969.71, 969.72, 969.73, 969.79, E85.42
ICD-10	F15.X
Naltrexone (oral or extended release)	
APCD Pharmacy NDC Codes	Extended-release naltrexone: 657570301, 63459030042<br><br>Oral naltrexone: 54868557400, 54569913900, 54569672000, 50090307600, 50090286600, 16729008101, 16729008110, 52152010502, 52152010530, 53217026130, 68084029111, 68084029121, 52152010504, 42291063230, 63629104701, 63629104601, 68115068030, 65694010010, 65694010003, 00904703604, 43063059115, 76519116005, 68094085359, 68094085362, 00185003930, 00185003901, 00406117001, 00406117003, 47335032688, 47335032683, 51224020650, 51224020630, 00555090201, 00555090202, 50436010501, 00056001170, 00056001130, 00056007950, 00056001122, 51285027502, 51285027501, 00056008050
Procedure Codes	Extended-release naltrexone: J2315, G073, HZ94ZZZ

eTable 6. Datasets used to Identify Homelessness in the Massachusetts Public Health Data Warehouse

Department of Mental Health
Department of Transitional Assistance
Women, Infants and Children Nutrition Program
Massachusetts Ambulance Trip Record Information System
Prescription Monitoring Program or HIV Incidence Data
Bureau of Substance Addiction Services licensed treatment program, self reported homelessness at time of admission
APCD or Acute Care Hospital Case Mix (ICD-9 or 10 code for homelessness)
Department of Housing and Community Development

© 2024 Kimmel SD et al. *JAMA Network Open.*

eTable 7. Zero-inflated negative binomial model results: Characteristics associated with any buprenorphine receipt and weeks treated with buprenorphine among individuals with serious injection-related infections, Massachusetts, July 1st, 2014 to December 31st 2019[1]

	Any Buprenorphine, Adjusted Odds Ratio (AOR)				Total Weeks on Buprenorphine, Incident Rate Ratio (IRR)			
	**AOR**	**LCL**	**UCL**	**P-value**	**IRR**	**LCL**	**UCL**	**P-value**
**Age**								
18-34	REF				REF			
35-49	0.89	0.77	1.03	0.13	1.14	1.05	1.24	0.003
50-64	0.62	0.52	0.73	<.001	1.09	0.98	1.21	0.12
**Sex**								
Female	0.94	0.83	1.06	0.31	0.98	0.91	1.05	0.56
**Race and Ethnicity**								
White Non-Hispanic	REF				REF			
Black non-Hispanic	0.74	0.59	0.93	0.01	0.92	0.79	1.07	0.27
Hispanic	0.85	0.71	1.02	0.08	1.00	0.89	1.12	1.00
American Indian/Other/Unknown	0.65	0.34	1.26	0.20	0.96	0.60	1.53	0.86
**Insurance**								
Commercial	REF				REF			
Medicaid	1.22	1.02	1.46	0.03	1.02	0.91	1.14	0.74
Medicare	1.09	0.88	1.34	0.43	0.91	0.80	1.04	0.16
Self-pay	0.98	0.66	1.46	0.94	1.06	0.84	1.34	0.61
Other	0.94	0.56	1.58	0.82	0.84	0.60	1.18	0.31
**Homelessness**	1.36	1.17	1.59	<.001	0.97	0.89	1.06	0.48
**Anxiety/Depression**	1.33	1.16	1.52	<.001	1.11	1.02	1.21	0.02
**Alcohol Use disorder**	0.99	0.86	1.13	0.83	1.06	0.98	1.15	0.13
**Stimulant Use disorder**	1.02	0.87	1.20	0.79	0.94	0.86	1.03	0.19
**Opioid prescription, prior**	0.64	0.56	0.73	<.001	0.86	0.79	0.93	0.00
**Benzodiazepine prescription, prior**	0.98	0.85	1.13	0.79	1.06	0.97	1.15	0.19
**Naloxone prescription, prior**	1.25	1.03	1.52	0.02	1.01	0.92	1.11	0.86
**Modified Elixhauser Score[2]**								
0	REF				REF			
1	0.95	0.77	1.18	0.66	1.10	0.97	1.25	0.13
2	0.90	0.73	1.13	0.37	1.12	0.99	1.27	0.08
3+	0.71	0.58	0.87	<.001	1.05	0.94	1.18	0.39
**MOUD, prior**								

© 2024 Kimmel SD et al. *JAMA Network Open.*

Buprenorphine	15.94	13.87	18.32	<.001	1.40	1.30	1.52	<.001
Methadone	0.60	0.51	0.72	<.001	0.84	0.74	0.95	0.01
Naltrexone (oral or extended-release)	1.03	0.79	1.36	0.82	0.92	0.78	1.08	0.29
No MOUD	REF				REF			
**Opioid overdose, previous 6 months**	1.00	0.84	1.19	0.98	0.90	0.81	0.99	0.03
**Disposition Site**								
Home/routine discharge	REF				REF			
Home with services	0.76	0.63	0.93	0.01	1.00	0.88	1.12	0.95
Transferred, acute care hospital	1.01	0.80	1.26	0.96	0.93	0.81	1.07	0.30
SNF/rehab	0.84	0.71	0.99	0.04	0.91	0.82	1.00	0.06
Patient Directed Discharge	0.97	0.81	1.18	0.78	0.81	0.73	0.91	0.00
Other	0.99	0.73	1.33	0.93	0.99	0.83	1.17	0.89
**Infection type**								
Endocarditis	1.56	1.32	1.85	<.001	1.03	0.93	1.13	0.61
Epidural abscess	1.19	0.93	1.51	0.16	1.01	0.87	1.17	0.86
Septic arthritis	1.39	1.14	1.69	0.001	0.92	0.82	1.04	0.17
Osteomyelitis	0.98	0.82	1.15	0.78	1.10	0.98	1.22	0.10
Bloodstream infection	REF				REF			

© 2024 Kimmel SD et al. *JAMA Network Open.*

eTable 8. Zero-inflated negative binomial model results: Characteristics associated with any methadone receipt and weeks treated with methadone among individuals with serious injection-related infections, Massachusetts, July 1st, 2014 to December 31st 2019[1].

	Any Methadone, Adjusted Odds Ratio (AOR)				Total Weeks on Methadone, Incident Rate Ratio (IRR)			
	AOR	LCL	UCL	P-value	IRR	LCL	UCL	P-value
**Age**								
18-34	REF				REF			
35-49	0.93	0.80	1.07	0.31	1.08	0.99	1.17	0.09
50-64	0.55	0.46	0.67	<.001	1.12	1.01	1.26	0.04
**Sex**								
Female	1.25	1.10	1.42	<.001	1.01	0.94	1.09	0.80
**Race and Ethnicity**								
White Non-Hispanic	REF				REF			
Black non-Hispanic	0.92	0.73	1.17	0.51	0.92	0.79	1.07	0.28
Hispanic	1.10	0.92	1.32	0.30	0.99	0.89	1.10	0.84
American Indian/Other/Unknown	0.44	0.19	0.98	0.05	1.17	0.69	1.97	0.56
**Insurance**								
Commercial	REF				REF			
Medicaid	1.97	1.60	2.43	<.001	1.02	0.90	1.16	0.75
Medicare	1.17	0.92	1.49	0.21	1.04	0.89	1.21	0.64
Self-pay	1.73	1.14	2.60	0.01	1.02	0.81	1.29	0.86
Other	0.43	0.20	0.92	0.03	1.01	0.61	1.69	0.96
**Homelessness**	1.31	1.12	1.53	<.001	0.87	0.79	0.95	0.0003
**Anxiety/Depression**	1.15	0.99	1.33	0.06	1.00	0.92	1.09	1.00
**Alcohol Use disorder**	0.87	0.75	1.00	0.05	0.92	0.85	1.00	0.05
**Stimulant Use disorder**	1.20	1.02	1.40	0.02	0.92	0.84	1.00	0.06
**Opioid prescription, prior**	0.68	0.59	0.78	<.001	0.98	0.91	1.07	0.69
**Benzodiazepine prescription, prior**	0.91	0.78	1.06	0.21	1.00	0.92	1.09	0.97
**Naloxone prescription, prior**	1.35	1.12	1.64	0.002	0.97	0.87	1.08	0.61
**Modified Elixhauser Score[2]**								
0	REF				REF			
1	0.93	0.75	1.16	0.54	1.04	0.92	1.17	0.55
2	0.92	0.73	1.15	0.46	1.09	0.96	1.24	0.18
3+	0.76	0.62	0.93	0.01	1.00	0.89	1.12	0.94

© 2024 Kimmel SD et al. *JAMA Network Open.*

**MOUD, prior**								
Buprenorphine	0.97	0.84	1.13	0.71	0.99	0.89	1.09	0.79
Methadone	31.55	26.69	37.31	<.001	1.66	1.53	1.79	<.001
Naltrexone (oral or extended-release)	0.88	0.67	1.16	0.37	1.00	0.83	1.20	0.99
No MOUD	REF				REF			
**Opioid overdose, previous 6 months**	1.04	0.87	1.25	0.63	0.99	0.89	1.11	0.90
**Disposition Site**								
Home/routine discharge	REF				REF			
Home with services	0.77	0.62	0.95	0.01	1.08	0.95	1.22	0.25
Transferred, acute care hospital	0.88	0.69	1.13	0.33	1.01	0.88	1.17	0.84
SNF/rehab	0.91	0.77	1.09	0.31	0.96	0.87	1.06	0.40
Patient Directed Discharge	0.91	0.75	1.11	0.35	0.88	0.79	0.98	0.02
Other	1.29	0.96	1.74	0.09	1.01	0.85	1.20	0.90
**Infection type**								
Endocarditis	1.34	1.12	1.59	0.001	1.05	0.95	1.17	0.33
Epidural abscess	1.26	0.98	1.62	0.07	1.01	0.88	1.17	0.88
Septic arthritis	1.60	1.30	1.96	<.0001	1.01	0.90	1.14	0.83
Osteomyelitis	1.13	0.94	1.35	0.20	0.99	0.89	1.10	0.86
Bloodstream infection	REF				REF			

© 2024 Kimmel SD et al. *JAMA Network Open.*

eTable 9. Zero-inflated negative binomial model results: Characteristics associated with any extended-release naltrexone receipt and weeks treated with extended-release naltrexone among individuals with serious injection-related infections, Massachusetts, July 1st, 2014 to December 31st 2019[1]

	**Any Extended-Release Naltrexone, Adjusted Odds Ratio (AOR)**				**Total Weeks on Extended-Release Naltrexone, Incident Rate Ratio (IRR)**			
	**AOR**	**LCL**	**UCL**	**P-value**	**IRR**	**LCL**	**UCL**	**P-value**
**Age**								
18-34	REF				REF			
35-49	0.52	0.39	0.68	<.001	1.24	0.98	1.56	0.07
50-64	0.29	0.19	0.44	<.001	1.02	0.68	1.52	0.94
**Sex**								
Female	0.88	0.68	1.13	0.31	1.06	0.85	1.30	0.62
**Race and Ethnicity**								
White Non-Hispanic	REF				REF			
Black non-Hispanic	0.81	0.48	1.36	0.42	1.04	0.66	1.66	0.86
Hispanic	1.26	0.87	1.84	0.22	0.58	0.41	0.81	0.001
American Indian/Other/Unknown	0.25	0.03	2.14	0.21	1.13	0.20	6.40	0.89
**Insurance**								
Commercial	REF				REF			
Medicaid	1.02	0.72	1.44	0.92	0.95	0.70	1.29	0.75
Medicare	0.37	0.21	0.65	<.001	1.51	0.90	2.53	0.12
Self-pay	0.70	0.24	2.04	0.51	1.11	0.56	2.21	0.76
Other	0.89	0.41	1.95	0.77	1.63	0.64	4.15	0.30
**Homelessness**	0.88	0.65	1.17	0.38	0.99	0.78	1.25	0.92
**Anxiety/Depression**	1.29	0.97	1.71	0.08	0.83	0.65	1.06	0.14
**Alcohol Use disorder**	1.05	0.80	1.37	0.72	0.96	0.76	1.21	0.71
**Stimulant Use disorder**	1.05	0.79	1.40	0.75	1.15	0.89	1.48	0.27
**Opioid prescription, prior**	0.87	0.65	1.16	0.34	0.89	0.70	1.12	0.31
**Benzodiazepine prescription, prior**	1.03	0.76	1.39	0.87	1.04	0.80	1.35	0.76
**Naloxone prescription, prior**	0.94	0.66	1.35	0.75	1.15	0.86	1.55	0.34
**Modified Elixhauser Score[2]**								
0	REF				REF			
1	1.00	0.70	1.43	0.98	1.20	0.89	1.61	0.24
2	0.61	0.41	0.90	0.01	1.32	0.95	1.85	0.10
3+	0.39	0.27	0.58	<.0001	1.67	1.19	2.35	0.00
**MOUD, prior**								

© 2024 Kimmel SD et al. *JAMA Network Open.*

Buprenorphine	0.92	0.69	1.23	0.59	0.78	0.61	1.00	0.05
Methadone	0.63	0.43	0.93	0.02	0.84	0.59	1.20	0.33
Naltrexone (oral or extended-release)	10.38	7.70	14.00	<.001	1.12	0.90	1.39	0.30
No MOUD	REF				REF			
**Opioid overdose, previous 6 months**	1.33	0.98	1.80	0.07	0.93	0.73	1.19	0.57
**Disposition Site**								
Home/routine discharge	REF				REF			
Home with services	0.51	0.30	0.87	0.01	0.91	0.57	1.47	0.71
Transferred, acute care hospital	0.91	0.56	1.50	0.72	0.72	0.47	1.13	0.15
SNF/rehab	1.03	0.74	1.43	0.87	0.83	0.63	1.11	0.21
Patient Directed Discharge	1.26	0.90	1.77	0.18	0.70	0.53	0.92	0.01
Other	1.60	0.94	2.74	0.09	0.60	0.38	0.94	0.03
**Infection type**								
Endocarditis	1.13	0.83	1.54	0.44	1.30	0.99	1.69	0.05
Epidural abscess	0.51	0.29	0.93	0.03	1.50	0.89	2.50	0.12
Septic arthritis	0.84	0.56	1.28	0.42	0.93	0.64	1.36	0.72
Osteomyelitis	0.85	0.59	1.23	0.40	1.13	0.81	1.56	0.47
Bloodstream infection	REF				REF			

© 2024 Kimmel SD et al. *JAMA Network Open.*
