# Supplementary material for: Medication for Opioid Use Disorder After Serious Injection-Related Infections in Massachusetts
Source: JAMA Netw Open. 2024 Jul 24;7(7):e2421740. doi: 10.1001/jamanetworkopen.2024.21740 (PMC11270137; doi:10.1001/jamanetworkopen.2024.21740)
Supplement: Supplement 2. — Data Sharing Statement [file jamanetwopen-e2421740-s002.pdf]

Data Sharing Statement

Kimmel. Medications for Opioid Use Disorder After Serious Injection-Related Infections in Massachusetts. *JAMA Netw Open*. Published July 24, 2024.
doi:10.1001/jamanetworkopen.2024.21740

Data
Data available: Yes
Data types: Other (please specify)
Additional Information: Data is available from the Massachusetts Public Health Data Warehouse with approval.
How to access data: https://www.mass.gov/public-health-data-warehouse-phd
When available: With publication

Supporting Documents
Document types: None

Additional Information
Who can access the data: Researchers whose proposed use of the data has been approved.
Types of analyses: Approved by the Massachusetts Department of Public Health
Mechanisms of data availability: After approval of a proposal
